# Prevalence and socioeconomic and geographical inequalities of household food insecurity in the Paris region, France, 2010

**DOI:** 10.1186/1471-2458-13-486

**Published:** 2013-05-20

**Authors:** Judith Martin-Fernandez, Francesca Grillo, Isabelle Parizot, France Caillavet, Pierre Chauvin

**Affiliations:** 1 University Pierre et Marie Curie-Paris 6, UMR-S 707, Paris, France; 2 INSERM, U707, Research team on the social determinants of health and healthcare, 27 rue de Chaligny, Paris, 75012, France; 3 INRA, U1303, Research team on diet and social science, 65 boulevard de Brandebourg, Ivry sur Seine, 94205, France; 4 CNRS, ERIS, Research team on social inequality, 48 boulevard Jourdan, Paris, 75014, France

## Abstract

**Background:**

Food insecurity (FI) is the situation where people do not have, at all times, access to sufficient, safe and nutritious food that meets their dietary needs for an active and healthy life. The objectives of this study were to estimate the prevalence of FI in the Paris area by using, for the first time in France, a specific FI questionnaire and to identify the characteristics of food-insecure households, taking into account a potential neighbourhood effect.

**Methods:**

This study is based on data from the third wave of the SIRS cohort study (a representative, population-based socioepidemiological study) that were analysed using a cross-sectional design. In 2010, 3000 individuals in the Paris metropolitan area (PMA) were interviewed. FI was investigated by means of the USDA’s HFSSM. We used stratified multilevel models across three household income categories to identify populations at risk for FI.

**Results:**

In 2010, 6.30% (95% CI = [4.99-7.97]) of the households in the PMA experienced FI (up to 13.59% in the most underprivileged neighbourhoods). About 2.50% of the households experienced severe FI and 2.85% of household living with an income above 1666 € experienced food insecurity, whereas the percentage raises to 23.38% among those living below the poverty threshold (<791 €). Depending on the income level, different household characteristics emerged as being associated with FI. In the poorest households, the presence of a child under 3 years of age was associated with an increased risk of FI (OR = 2.11; p = 0.03). Among higher-income households, the household composition appeared to be strongly associated with FI.

**Conclusion:**

FI exists in several social groups in France. Its prevalence in the most underprivileged households should be considered an indicator of vulnerability, which could permit targeted social assistance policies.

## Background

In the present global financial crisis, health-related and population-based indicators of poor living conditions are more important clues in social epidemiology than ever for better defining priorities in public health and social policies. Food insecurity (FI) is defined as the situation where people do not have, at all times, physical, social and financial access to sufficient, safe and nutritious food that meets their dietary needs for an active and healthy life [1]. Food insecurity is a potential risk factor for poor health [2,3], unhealthy eating patterns [4-6], chronic disease [7] and mental distress [8]. It is a well-known aspect of living in underprivileged conditions in certain countries, such as the United States and Canada, where various studies have been conducted in the last 20 years [9-14]. However it is much less known and studied in France in the field of social epidemiology and poverty research [10,15,16]. The present economic crisis is having a strong impact on employment and poverty in France [17], and it is generally known that the most vulnerable people are particularly affected by price changes and financial shocks [18]. The lack of an estimate of FI in France is particularly critical, and information is not clear as to whether or not the French welfare system is protecting underprivileged populations against FI (and to what extent). For the record, this welfare system consists of a repository of interdependent public retirement, unemployment and health insurance plans but also involves the provision of a minimum social income and social housing [19]. In France, there is no public aid dedicated to food (either in kind or in cash). There are only non-governmental food-aid organizations, which provide an indication of the scope of the problem and which have repeatedly alerted the public and policy-makers to the increasing number of aid recipients. For instance, in 2010, 185 million meals were served by food banks and 740,000 people visited these organizations in all of France, while 663,000 visited them in 2008. In 2010, in the Paris metropolitan area, 91,000 people visited a food bank every month and 11.2 million meals were served [20]. A food sufficiency question was included for the first time in two French national surveys conducted between 2006 and 2007 [21] and in 2008 [22]. They corroborate the existence of such a problem in France, even if they used a very brief indicator of FI that is no longer used at the national level in other Western countries. Since these indicators referred broadly to FI, a widely used and very detailed tool seemed necessary for describing this phenomenon with greater accuracy, which would make it possible to assess FI severity and make international comparisons. The objectives of this study, which was conducted in the Paris metropolitan area in 2010, were to estimate the prevalence of FI and to study the main household (HH) socioeconomic factors and neighbourhood inequalities [23] associated with FI.

## Methods

### Study design and sample

This study is based on a cross-sectional analysis of data collected in 2010 in the SIRS (a French acronym for “health, inequalities and social ruptures”) cohort study among a representative sample of French-speaking adults in the Paris metropolitan area (Paris and its suburbs, a region with a population of 5.18 million). Since 2005, the objective of the cohort study has been to investigate the relationships between individual, household and neighbourhood social characteristics, and health-related conditions. A 3-level random sample was constructed at inclusion in 2005. First, 50 census blocks called “IRISs” (a French acronym for “blocks for incorporating statistical information”, which constitute the smallest census unit areas in France whose aggregate data can be used on a routine basis, with about 2,000 inhabitants each) were randomly selected using a stratification based on their socioeconomic type and their being or not being labelled as “underprivileged areas” in public (government) policies. The lower-income neighbourhoods were overrepresented. Next, 60 households (HHs) were randomly chosen from a complete list of HHs within each selected IRIS. Lastly, one adult was randomly selected from each HH by the birthday method [24]. In 2010, in the third wave, 47% of the respondents were reinterviewed face-to-face at home (2.6% were deceased, 1.8% were too sick to answer our questions, 2.7% were absent during the survey period, 13.9% had moved out of the 50 surveyed IRISs, 18.4% declined to participate, and 13.4% were lost to follow-up). Their sex ratio and mean age were similar to those who were not reinterviewed. The individuals lost to follow-up were younger and wealthier than the others, but neither their health status nor the type of IRIS of residence was different. Those absent during the survey period had a lower socioeconomic status and were mostly immigrants. The individuals in each IRIS who were not reinterviewed in 2010 were replaced by a random procedure similar to the one used in 2005, up to a final sample size of 60 adults interviewed per IRIS. The refusal rate among the newly contacted people was 29% (the same as in 2005). This cohort study was approved by the French privacy and personal data protection authority (Commission Nationale de l’Informatique et des Libertés).

### Food security

FI was measured by the Household Food Security Scale Measure (HFSSM), a scale created by the U.S. Department of Agriculture (USDA) [12], which was used for the first time in France, in the third wave of the SIRS cohort study in 2010. It had been translated into French (the translation was modelled after the French translation used in Quebec for the 2004 cycle of the Canadian Community Health Survey) [25]. This tool measures in qualitative and quantitative terms of compromises in food intake during the last 12 months (e.g., running out of food or money to buy food, skipping meals, and buying cheaper food) with declining household economic resources [26]. Originally, the HFSSM questionnaire included a preliminary question (see below) and 18 items (10 adult-referenced and 8 child-referenced). To shorten interview time, we chose to reduce the instrument to 13 questions by removing the last five child-referenced questions (which were asked only at HHs with children under 18 years of age that had experienced the severest degree of FI). In other words, we kept all the adult-referenced questions and only three of the child-referenced questions (see Additional file 1). Such a reduction did not affect the measure of FI at the HH level, since all the household-related questions were still asked in the order determined by the USDA [27]. For the purposes of this paper, we excluded the child-referenced questions. A single score (being the total number of affirmative responses — yes or sometimes/often — to the 10 remaining adult-referenced questions) was calculated, as has been done in other studies [25,27,28]. Ranging from 0 to 10, this score was divided into three categories defined by the usual thresholds: food security (score < 3), low food security (score = 3–5) and very low food security (score > 5) [27,29]. According to Bickel et al. [27,29], low food security (FS), formerly referred to a “food insecurity without hunger” [29], is the condition where “Food insecurity is evident in household members’ concerns about adequacy of the household food supply and in adjustments to household food management, including reduced quality of food and increased unusual coping patterns. Little or no reduction in members’ food intake is reported”. Very low FS, formerly referred to as “food insecurity with hunger”, is the situation where “the food intake of household members was reduced and their normal eating patterns were disrupted because the household lacked money and other resources for food” [30]. We used this classification for the prevalence and univariate analysis, and a dichotomous variable (food-secure/food-insecure - i.e. low food security and very low food security -) for further logistic regressions.

### Covariates

Since FI was assessed at the HH level, all the covariates used in this research were captured and measured at the HH level as well. HH composition was determined by means of a 5-category variable based on that used by the French National Bureau of Statistics: single-family HH, two-or-more family HH, single-parent family, single-person HH, and unrelated-persons HH^a^. We also noted the presence of any children in the HH, and, in multivariate analysis, we distinguished HHs with at least one child under the age of 3 years (i.e., the school starting age in France).

As regards HH demographics and socioeconomic status, the following variables were considered: the HH head’s gender, age (in three categories), education level (in three categories), occupational status (in six categories: active worker, student, unemployed, retired, homemaker, and disabled person) and socio-occupational category (a 6-category variable based on the French National Bureau of Statistics’ classification: upper white-collar, tradespeople/salespersons, middle white-collar, lower-white collar, blue-collar and never worked, with retired and inactive persons classified according to their last job).

HH monthly income was calculated as the total HH income divided by the number of people in the consumption unit (CU) on the basis of the OECD scale [31] in order to take household composition into account^b^. It was used as a continuous numerical variable (expressed in hundreds of €/CU), then as a stratification variable (due to interactions discussed below) in three categories: < 791 €/CU (the French poverty threshold, i.e., 50% of the median French income), [791–1166 €/CU] (1166 €/CU marking the first quartile of the sample’s income distribution) and > 1166 €/CU. We also took note of the income source (welfare versus other) and whether the household was in social housing at the time of the study.

To study the sociogeographical inequalities in FI, we also took into account the socioeconomic status of the residential neighbourhood of each HH (this neighbourhood being defined as the IRIS of residence; see above). Neighbourhood status was assessed on the basis of a 3-category variable: the most underprivileged, working-class, or middle- or upper-class. The most underprivileged neighbourhoods were labelled as such by French national urban policies in order to identify the poorest neighbourhoods that might benefit from special urban, social and economic positive action. The “working-class” and “middle- or upper-class” categories correspond to an existing typology for the Paris region developed and validated by E. Preteceille and based on the residents’ prevailing socio-occupational categories [32].

### Statistical methods

FI prevalence was calculated for the entire study population and within subgroups. These estimates, as well as the univariate analyses, were weighted to take into account the complex sample design and the post-stratification adjustment for age and gender according to the 2008 general population census data. Chi-square tests were used to compare proportions.

Since FI was strongly associated with HH income and since HH income interacted with other characteristics, we performed a stratified analysis by HH income category to identify the factors associated with FI within each HH income stratum. Multilevel logistic regression models were fitted to identify HH socioeconomic and residential neighbourhood characteristics associated with FI. All the covariates were introduced into a multilevel model adjusted for HH head age and gender and were then backward-selected to keep those significantly associated with FI in each income stratum. Residential neighbourhood status was forced into the models. All the analyses were performed with SPSS 19 and Stata 11.

## Results

### Prevalence

During the previous 12 months, 6.30% (95% CI = [4.99-7.97]) of HHs had experienced FI: 3.90% (95% CI = [3.07-4.87]) low FS and 2.40% (95% CI = [1.66-3.61]) very low FS. Extrapolated to the entire population in the Paris metropolitan area, these percentages yield an estimated 326,000 adults who were living in food-insecure HHs, with 124,200 of them living in HHs with very low FS.

### Univariate results

Various characteristics were associated with FI in univariate analysis (Table 1). The prevalence of FI decreased with HH head age, from 11.30% (95% CI = [7.24-17.20]) for HH heads aged 18 to 29 years to 3.34% (95% CI = [2.08-5.34]) for HH heads aged 60 years and over. It should be noted that very low FS was rare among households headed by older persons (0.50%).

**Table 1:** **Prevalence**^**a**^** of household food insecurity according to various demographics and socioeconomic and neighbourhood characteristics, Paris metropolitan area, 2010**

		**Low FS**			**Very low FS**			**Total food insecurity**		
	**Weighted population**^**a**^	**Prevalence**^**a**^	**95% CI**^**a**^	***p***	**Prevalence**^**a**^	**95% CI**^**a**^	***P***	**Prevalence**^**a**^	**95% CI**^**a**^	***P***
Total population	3006	3.90%	[3.07-4.87]		2.40%	[1.65-3.61]		6.30%	[4.99-7.96]	
Household (HH) type										
Single-person HH	572	3.16%	[1.95-5.08]	<0.001	2.42%	[1.46-3.98]	<0.001	5.58%	[4.03-7.67]	<0.001
Single-family HH	1972	2.56%	[1.76-3.72]		1.61%	[0.87-2.98]		4.17%	[2.81-6.16]	
Two-or-more family HH	75	13.62%	[5.86-28.54]		8.85%	[4.18-17.80]		21.47%	[12.73-36.55]	
Single-parent family	279	12.15%	[8.28-17.47]		4.97%	[3.03-8.04]		17.11%	[12.76-22.57]	
Unrelated-persons HH	163	2.97%	[0.63-12.89]		5.05%	[1.49-15.80]		8.02%	[3.12-19.12]	
Number of children^b^ in the HH										
None	1867	2.89%	[2.01-4.16]	<0.001	1.82%	[1.18-2.83]	0.006	4.71%	[3.58-6.23]	<0.001
1 or 2	892	5.16%	[3.60-7.21]		3.03%	[1.70-5.28]		8.18%	[5.70-11.44]	
3 or more	247	6.71%	[4.10-10.82]		5.06%	[2.43-10.23]		11.77%	[7.49-18.03]	
HH head's gender										
Male	2286	3.46%	[2.62-4.54]	0.046	2.27%	[1.44-3.62]	0.332	5.73%	[4.26-7.70]	0.022
Female	719	5.15%	[3.66-7.31]		2.92%	[1.87-4.61]		8.07%	[6.23-10.56]	
HH head’s age										
18-29	314	6.43%	[3.63-11.14]	0.02	4.87%	[2.16-10.62]	<0.001	11.30%	[7.24-17.20]	<0.001
30-59	1872	3.88%	[2.89-05.20]		2.90%	[1.83-4.56]		6.78%	[5.15-8.88]	
60 or over	820	2.85%	[1.71-04.71]		0.50%	[0.21-1.19]		3.34%	[2.08-5.34]	
HH head's socio-occupational category										
Never worked	87	3.87%	[0.58-21.66]	<0.001	3.40%	[1.00-10.96]	<0.001	7.27%	[2.39-20.08]	<0.001
Upper white-collar	1066	1.29%	[0.58-2.84]		0.56%	[0.12-2.58]		1.85%	[0.93-3.66]	
Tradespeople/ salespersons	209	2.79%	[0.81-9.21]		1.42%	[0.41-4.79]		4.21%	[1.71-9.97]	
Intermediate white-collar	360	3.52%	[1.67-7.28]		2.15%	[0.93-4.92]		5.67%	[3.30-9.58]	
Lower white-collar	893	6.10%	[4.55-8.14]		4.94%	[3.17-7.61]		11.04%	[8.70-13.91]	
Blue-collar	335	6.93%	[4.44-10.65]		2.74%	[1.46-5.07]		9.67%	[6.65-13.84]	
HH head's education level										
Tertiary	1580	2.59%	[1.57-4.29]	<0.001	1.33%	[0.71-2.40]	<0.001	3.92%	[2.69-5.67]	<0.001
Secondary	1053	4.56%	[3.50-5.86]		3.70%	[2.35-5.75]		8.26%	[6.41-10.51]	
None or primary	352	7.69%	[4.75-12.38]		3.70%	[1.99-7.19]		11.40%	[7.37-17.66]	
HH monthly income (€/CU^c^)										
>1666	2211	2.00%	[1.25-3.16]	<0.001	0.90%	[0.45-1.55]	<0.001	2.85%	[1.87-4.28]	<0.001
[791–1166]	439	6.60%	[4.57-9.32]		3.40%	[1.99-5.57]		10.00%	[7.40-13.12]	
<791 (poverty threshold)	356	12.10%	[8.68-16.89]		11.30%	[7.27-17.29]		23.38%	[17.57-30.81]	
Source of income										
Welfare benefits	235	17.63%	[12.58-24.15]	<0.001	9.33%	[5.72-14.87]	<0.001	26.96%	[20.91-34.00]	<0.001
Other	2738	2.74%	[2.04-3.66]		1.84%	[1.22-2.77]		4.58%	[3.52-5.93]	
Resident of social housing										
No	2244	2.53%	[1.73-3.66]	<0.001	1.47%	[0.86-2.49]	<0.001	3.99%	[2.86-5.55]	<0.001
Yes	762	7.82%	[6.06-10.03]		5.33%	[3.58-7.87]		13.15%	[10.19-16.80]	
Neighbourhood socioeconomic status										
Underprivileged	448	8.91%	[6.61-11.70]	<0.001	4.68%	[2.56-8.45]	<0.001	13.59%	[9.25-19.29]	<0.001
Working-class	489	6.35%	[4.66-8.61]		2.05%	[0.96-4.56]		8.40%	[5.55-12.62]	
Middle- or upper-class	2069	2.22%	[1.35-3.61]		2.03%	[1.14-3.64]		4.25%	[2.86-6.30]	

^a^ Weighted.

^b^ Under 18 years old.

^c^ Consumption unit.

Naturally, income was found to be dramatically associated with FI. The prevalence of very low FS among the HHs living under the poverty threshold was almost 12 times higher than that among HHs with a monthly income greater than 1666 €/CU (p < 0.001). The prevalence of FI was highest among welfare recipients: one-fourth (26.96%; 95% CI = [20.91-34.00]) of them and almost 10% (9.33%; 95% CI = [5.72-14.87]) of them had experienced low or very low FS, respectively. As for the individuals who were living in social housing, the prevalence of FI was 3 times higher than in those who were not: 13.15% (95% CI = [10.19-16.80]), with 7.82% (95% FI = [6.06-10.03] and 5.33% (95% CI = [3.58-7.87]) of them experiencing low or very low FS, respectively.

Although FI was surprisingly present in all the socio-occupational groups, including the higher ones, it was more prevalent in the blue-collar and lower white-collar categories (11.04% and 9.67%, respectively). As well, while the average figure for the overall FI prevalence was observed in food-insecure households headed by a person who had never worked, almost half of them had experienced very low FS. We also observed a significant gradient according to the education level (p < 0.001). The FI prevalence also differed according to household type and composition. Single-family HHs and HHs without children had a lower FI prevalence (4.17%, 95% CI = [2.77-6.12] and 4.71%, 95% CI = [3.58-6.23], respectively), while two-or-more-family HHs and HHs with 3 or more children had a higher FI prevalence. Single-parent families were also more affected by FI (17.11%, 95% CI = [12.76-22.57]), and 4.97% (95% CI = [3.03-8.04]) of them had experienced very low FS. Unrelated-persons HHs seemed to be a special case, since they had a higher prevalence of very low FS than low FS (5.05% vs. 2.97%, respectively), even if the differences were not significant. The prevalence of FI also differed according to HH head gender. Although female-headed HHs had a higher (but not significant) prevalence of FI, they were very close to male-headed HHs in the specific case of very low FS. Lastly, the FI prevalence was 3 times higher in the underprivileged neighbourhoods than in the middle- or upper-class ones (respectively, 4 times higher for low FS and 2 times higher for very low FS).

### Multilevel analysis

Multilevel analyses were stratified into three income groups (< 791; [791–1166]; >1166 €/CU). It is worth noting that HHs of the poorest group were headed more often by a woman (31.4% vs. 21.3% for those with an income above 1166€, *p* < 0.001) or by an unemployed adult (22.9% vs. 1.7% for the richer HHs, *p* < 0.001). The poorest HHs had also more often 3 children or more (17.0% vs. 5,4% for the richer HHs, *p* < 0.001).

The three models (Table 2) report the likelihood of having experienced FI in the previous 12 months and show different associations according to HH income level.

In the poorest category, further adjustment for HH income as a continuous variable was not significant. On the contrary, HH income was a significant protective factor in the top two categories: OR = 0.70 (95% CI = [0.53-0.91]) for a HH income between 791 and 1166 €/CU and OR = 0.88 (95% CI = [0.83-0.94]) for a HH income above 1166 €/CU.

After adjustment for HH income, a few characteristics remained significantly associated with FI in one or more income groups. Having a child under the age of 3 years doubled the risk of FI among the poorest HHs (OR = 2.11; 95% CI = [1.08-4.12]). This association was weaker in the higher-income groups and no longer significant. The only characteristic significantly associated with FI in all three income groups was HH composition. Considering single-family households as the reference, several types were more likely to experience FI. In the highest-income groups, two-or-more-family households were significantly associated with FI (OR = 5.58 for the middle-income group and OR = 5.36 for the lowest group). This association was also observed in the poorest-HH group (OR = 2.24) but was not statistically significant (*p* = 0.16). Single-parent families were more at risk in all three income groups, and the OR estimates were even higher – if not significant – in the top two income groups (OR = 5.60 and 5.85 for middle-income HHs and HHs with a monthly income above 1166 €/CU, respectively) than among the poorest HHs (OR = 2.89). Single-person households were also significantly and strongly at greater risk than the reference HH type in the top two income groups (OR = 3.28 for the middle-income group and OR = 3.43 for the highest). Lastly, unrelated-persons households were significantly associated with a higher risk of FI in the highest-income group only (OR = 6.57).

In multivariate analysis, once adjusted for the previous HH characteristics, neighbourhood socioeconomic status was no longer significantly associated with FI. Actually, only the association with underprivileged neighbourhoods in the [791–1166 €/CU] income group was at the limit of significance (OR = 2.15; 95% CI = [0.96-4.80]).

Due to the small sample size, other characteristics were not statistically significant, but some observed associations may be mentioned here, even if they were not significant. For instance, the fact of women heading households might have a protective effect in all the income groups. Also, HH head age and occupation appeared to be associated with FI. In the higher-income groups, the likelihood of being food-insecure seemed to show an increasing trend as household head age decreased. As for HH head occupation, no trend was particularly observable, except for disabled persons, who seemed to be more prone to FI, this with a strong OR (34.98), although the confidence interval was extremely broad.

**Table 2:** **Household (HH) demographics and socioeconomic and neighbourhood characteristics associated with food insecurity (FI), multivariate, multilevel analysis stratified by HH monthly income group, Paris metropolitan area, 2010** (^a^)

	**Household income ≤ 791 € (poverty threshold for household income per CU) n=371**			**Household income [791–1166 €] n=542**			**Household income >1166 € n=2093**		
	**% FI**^**a**^	**aOR**	**95% CI**	**% FI**^**a**^	**aOR**	**95% CI**	**% FI**^**a**^	**aOR**	**95% CI**
**HH income per CU/100 €**									
		0.90	[0.75-1.08]		**0.70**	**[0.53-0.91]**		**0.88**	**[0.83-0.94]**
**HH type**									
Single-family HH	22.53	*Ref.*	-	8.20	*Ref.*	-	1.35	*Ref.*	-
Two-or-more family HH	47.06	2.24	[0.73-6.87]	26.67	**5.58**	**[1.34-23.24]**	7.69	**5.36**	**[1.09-26.45]**
Single-parent family	34.62	**2.89**	**[1.10-7.62]**	25.69	**5.60**	**[2.05-15.32]**	9.46	**5.85**	**[2.19-15.59]**
Single-person HH	22.22	1.32	[0.56-3.10]	12.50	**3.28**	**[1.20-8.96]**	3.48	**3.43**	**[1.56-7.54]**
Unrelated-persons HH	13.64	0.79	[0.19-3.32]	0.00	NC	.	7.04	**6.57**	**[2.13-20.25]**
**Presence of children < 3 years of age**									
No	22.26	*Ref.*	-	12.50	*Ref.*	-	2.84	*Ref.*	-
Yes	42.62	**2.11**	**[1.08-4.12]**	16.13	1.36	[0.58-3.16]	3.25	1.17	[0.42-3.24]
**HH head's gender**									
Male	26.09	*Ref.*	-	10.61	*Ref.*	-	2.31	*Ref.*	-
Female	24.82	0.55	[0.24-1.26]	16.51	0.65	[0.28-1.54]	4.21	0.72	[0.35-1.45]
**HH head's age**									
60 or over	18.52	*Ref.*	-	7.88	*Ref.*	-	1.18	*Ref.*	-
30-59	27.73	0.65	[0.25-1.70]	14.94	1.73	[0.28-10.77]	3.57	2.56	[0.64-10.21]
18-29	26.47	0.86	[0.22-3.41]	16.33	2.55	[0.33-19.86]	6.30	3.02	[0.61-14.96]
**HH head's occupation**									
Active worker	25.17	*Ref.*	-	15.18	*Ref.*	-	3.19	*Ref.*	-
Unemployed	31.87	1.33	[0.71-2.52]	15.91	1.05	[0.39-2.83]	7.89	1.64	[0.46-5.86]
Student	12.50	0.46	[0.07-2.90]	6.67	0.35	[0.03-4.01]	12.50	2.36	[0.41-13.42]
Retired	14.93	0.50	[0.17-1.49]	7.84	0.95	[0.14-6.38]	1.35	1.07	[0.27-4.26]
Homemaker	30.43	1.01	[0.33-3.05]	0.00	NC	.	7.14	1.38	[0.16-11.96]
Disabled	42.86	2.49	[0.86-7.22]	30.77	2.70	[0.61-11.95]	50.00	**34.98**	**[3.64-336.16]**
**Neighbourhood socio economic status**									
Middle- or upper- class	21.00	*Ref.*	-	8.57	*Ref.*	-	2.01	*Ref.*	-
Working-class	22.73	0.98	[0.46-2.08]	11.36	1.35	[0.57-3.19]	3.42	1.05	[0.51-2.18]
Underprivileged	30.43	1.30	[0.63-2.67]	18.32	2.15	[0.96-4.80]	5.41	1.70	[0.91-3.18]
**Between-area variation**		**Variance**	**SE**		**Variance**	**SE**		**Variance**	**SE**
		0.10	0.19		0.23	0.23		0.00	0.00

^a^ Unweighted.

## Discussion

The aim of the present study was to estimate the prevalence of food insecurity in the Paris area, using a standardised instrument that provides more precise indications and a more precise definition of food insecurity (and of its severity), thanks to the collection of declarative data on a set of lived experiences. Our study was the first one to have used this instrument in France, and it had the attribute of being population-based and representative of the Paris metropolitan area.

We found an overall FI prevalence of 6.30%, with a prevalence of very low FS of 2.40%. In this study, we identified several characteristics of HHs that had experienced FI within the previous 12 months. We found a higher prevalence of FI among HHs receiving welfare, two-or-more-family HHs and, of course, the poorest HHs. On the other hand, the prevalence was particularly low among HHs headed by an upper-white-collar worker or an elderly person.

Once adjusted for income, certain determinants were significantly associated with FI. First, the effect of sociodemographic variables (HH head age, gender and education level), economic indicators (source of income and residing in social housing) and contextual variables that was observed in univariate analysis disappeared after adjustment for income, which may be evidence of its strong impact. Second, the presence of a child under 3 years of age in the HH and being a single-parent family both remained associated with FI in the poorest group. This raises questions in the French context, where one might expect welfare policies to be specifically directed at single-parent families and at preventing children from experiencing food insecurity. Third, in the two groups above the poverty threshold, HH family composition was the most important determinant, after adjustment for income level. Indeed, when compared to single-family HHs, all other types of HHs were at higher risk for FI, which might be due to the additional socioeconomic constraints experienced by these families.

These results have some limitations. First, because of this study’s cross-sectional design, we cannot conclude that a particular family composition causes food insecurity, but rather that it only describes certain family types that are especially at risk. Second, it should be mentioned that our version of the HFSSM questionnaire contained fewer questions regarding child FI. However, this had no impact on our estimate, since we analysed FI among adults only and separately, as did a Canadian study previously [25]. One limitation of this study is the sample size, which may have sometimes resulted in a lack of strength in our analysis, but the punctual estimate (i.e., the OR estimate) can give an indication of the kind of association observed. A limitation of this study lies on the fact that our sample excluded homeless (population estimated in 2010 at almost 21 200 in Paris metropolitan area [33]) or non-French speaking people, who are certainly a population that may experience food insecurity. These exclusions may induce an under-estimation of the food insecurity phenomenon. Another limitation (common to all surveys that use this tool) is the failure to investigate all the dimensions of food insecurity as originally defined (particularly the social acceptability of food acquisition). Also, response bias may have resulted from the shame attached to FI, which may therefore have been underreported by the interviewees and have led to an underestimation of the prevalence of FI. Lastly, only one person in the HH answered the questionnaire, which was used to estimate FI for the entire HH. However, since 99% of the respondents were the HH head or his/her partner, they had a good knowledge of the situation in the HH. While the use of the HFSSM has been discussed in several studies and reports [34,35], we think that it is particularly useful for describing the situation in France because it enables one to estimate different ranges of FI severity and make international comparisons.

Two other indicators of FS (see Additional file 2) had been used previously in a secondary analysis of the Individual and National Food Consumption Survey (INCA 2)^c^ and in the Health and Nutrition Barometer (BSN)^d^ . In our survey, the FI prevalence was much lower than the prevalence of food insufficiency, as it can be estimated by applying the INCA 2 and BSN instruments to our study population: 6.30% for FI versus a food insufficiency prevalence of 20.9% and 10.5% in the BSN and INCA 2, respectively. This difference can be explained by the conceptual differences captured by these tools, for the HFSSM has a narrower definition of food hardship than the other two instruments.

The prevalence rate found in our study also seemed to be lower than the rates estimated with the same questionnaire in other Western countries. For example, the FI prevalence was found to be 14.5% in the U.S. in 2010 [36] and 7.7% in Canada in 2007–2008 [37]. Of course, these national prevalence rates were not directly comparable with our results for the Paris metropolitan area. However, similar or even greater differences were observed when we compared our results with FI prevalence rates for the main cities in the U.S. (according to 2003 Office of Management and Budget delineation), where the annual American food insecurity study reported an FI prevalence of 17% (with 10.7% of the urban population experiencing low food security and 6.3% very low food security) [36]. In the Montreal Health Region in 2008, 9.0% of HHs were living in FI (6.2% in low food security and 2.7% in very low food security) [38]. The fact that the FI prevalence is so much lower in the Paris metropolitan area may be due to its socioeconomic characteristics, for this region is known to be the wealthiest in France, and Paris is the second wealthiest city in the European Union (EU), after London [39]. But this may not be the only explanation, since the Paris region as a whole ranked 7th among EU regions in 2008 (as determined from the regional GDP per inhabitant by EUROSTAT) and is the region in France with the greatest social disparities [40]. It may also be due to the national context of the French welfare state’s (still) generous safety net. In the mid-2000s, the OECD estimated that the income poverty rate, which is based on 50% of the median income after taxes and transfers was 7.2%, 11.4% and 17.0% in France, Canada and the USA, respectively [41].

Consistent with the findings of studies carried out in the USA [30,42,43], Canada [9] and England [44], our study found that the prevalence of FI increased as HH income decreased. Moreover, it confirmed that in France, too, household composition is a major factor for food insecurity, as it is in Canada [37] and in the U.S. [45], where single-family households are less food-insecure (especially when there are no children). In both of these countries [9,30], households with children have a higher FI prevalence, and single-parent families are also more at risk, specifically, HHs with a single mother. This latter situation was observed in our study as well, in which single-parent families (most of which were headed by women) were at higher risk for FI in all income subgroups. In France, the specific family allowance for single parents ceased to exist as such and was included in a new, comprehensive allowance for the poor (which, in practice, is not very easy to obtain) right before the survey. As regards the association with education level, the gradient we observed was consistent with findings in the U.S. [45] but differed from the Canadian results [37], which did not seem to follow this pattern (perhaps because the proportion of the Canadian population with a tertiary education is particularly high).

Our results highlight some important points that may indicate specific vulnerabilities that characterise certain family situations and advocate for specific public policies targeted at these households (for example, special attention from a social worker). First, for the poorest households, we mentioned above the alarming situation regarding single-parent families and HHs with a preschool child. In France, in 2009, 4.5 million people were under the poverty threshold used in our models [46]. A recent study described the effect of a birth on the HH standard of living [47] and showed how the existing child welfare benefits may only partially offset the expenses, especially in terms of the impact on the occupational activity - and income - of one of the adults in the HH (and the only adult in single-parent families). These findings are worrisome, given the literature on the impact on children of living in a food-insecure HH [48-51] and the fact that food-insecure adults may compromise their nutrition to maintain that of their children [6,52]. Indeed, children’s nutrition, dietary intake and weight are affected, but this situation also has social, emotional and health (physical and mental) consequences [48-51,53-55].

In our study, HH composition appeared to be a more differentiating factor than HH head gender. Although female-headed HHs had a higher prevalence of FI, in multivariate analysis, they were not at greater risk. Since we stratified across income categories and adjusted for income and family composition, our results suggest that the greater vulnerability of female-headed HHs may be due to their worse poverty or their specific family composition. Indeed, previous findings in the literature [9,14,56] point to single-mother households being at greater risk for FI. On the other hand, the greater vulnerability of single-parent families must be due to factors other than the parent’s gender or income (which were not taken into account in the models), such as the proportion of the HH budget available for food (after the other expenses, such as daycare, are paid) and the unavailability to shop, which means not being able to prepare food at a lower cost.

A significant finding of our study was the high prevalence of FI among two-or-more-family HHs. They may be at greater risk for FI because of the number of adults and, therefore, the large quantity of food required. In the Paris area, they are mostly immigrant households and consequently more likely to be in underprivileged and precarious conditions [57]. Unfortunately, we could not test this hypothesis, since we did not know the immigration status of each member of the HHs. In the case of single-person and unrelated-persons households, we can assume, as we did for single-parent families, that the income variable used for our adjustment (before-tax total income per CU) conceals a lower disposable income than that of the reference (single-family) HH, once all the compulsory, nonreducible expenses are paid. A last result concerns the specific situation regarding the disabled, who were at greater risk for FI in all three income groups (even if the risk was significant only in the lowest group, with an extremely broad confidence interval). In France, the disabled have, on average, a particularly critical and low socioeconomic status [58] (their minimum net income guaranteed by Social Security is considerably below the poverty threshold).

## Conclusion

This study has highlighted a strong indicator of the social, health-related vulnerability of HHs in the Paris metropolitan area. It is quite remarkable, in a welfare state like France, where most of the public assistance to the poor, the disabled, the unemployed, single parents and others is provided in cash (through various forms of social assistance or benefits), not in kind, that a significant proportion of them are experiencing FI (26% of all the recipients of one of the various forms of minimum income benefits, 13% of people living in social housing or in underprivileged neighbourhoods labelled as such by public policies, etc.), with no public program or policy being debated or discussed at the national level to address this situation. Notwithstanding the fact that the literature has extensively showed that a sufficient, diversified and healthy diet is primarily determined by income [59,60], French minimum income guaranteed by social assistance (approx. 700 €/CU/month, depending on the benefits considered) remains under the poverty threshold, i.e. at a level that is barely incompatible with an healthy diet (whose cost was estimated to be around 3.5 €/day and per person in 2006 [61].

In France, food aid is mainly the responsibility of non-governmental organizations and local initiatives which have recently reported a 40% increase in demand since 2008. This is occurring at the same time as a drastic reduction in European subventions, with a 40% decrease announced for the 2014–2020 period. The population who use food aid is quite well characterized in France thanks to the Abena survey performed in 2004–2005 [62,63]. Our results show individuals who use food aid constitute a small fraction of all food insecure people (as for other social services, many people in need may refrain from attending food aid services for different reasons, such as feelings of shame or stigma). The use of the HFSSM instrument in a national, representative survey in France seems necessary in order to obtain an internationally comparable estimate of the national FI prevalence. Our results emphasise the need, in the context of the present financial crisis, to discuss food aid and vulnerability at the national level and to present specific public policies aimed at the most vulnerable households, most of which are currently known to welfare programs.

## Endnotes

^a^Single family HH = one couple with or without kid, can also incorporate other persons (like a grand-parent).

Two-or-more family HH = Two-or-more couples with ou without kid, can also incorporate other persons (like a grand-parent).

Single parent family = HH with one parent and kid, can also incorporate other persons (like a grand-parent). Unrelated-persons = persons living together but who do not share a family link.

^b^The method used in this survey is the one developed by the OECD, that assigns a weight to each member – or consumption units (CU) - of the household to allow for comparisons between households of different sizes and compositions. The CU scale is the following one:

- 1 CU for the first adult in the household;

- 0.5 CU for the other persons aged 14 years or older;

- 0.3 CU for the children under 14 years.

^c^Which was conducted among a representative sample of the French population in 2006–2007 (n = 2624).

^d^A telephone survey of a random sample of the entire French population in 2008 (n = 3444), to characterise food insufficiency in the French context.

## Competing interest

The authors declare that they have no computing interests.

## Authors’ contributions

JMF: has made substantial contributions to conception and design, acquisition of data, analysis and interpretation of data and has been involved in drafting the manuscript and revising it critically for important intellectual content. FG: has made substantial contributions to conception and design, or acquisition of data, analysis and interpretation of data and has been involved in drafting the manuscript and revising it critically for important intellectual content. IP: has been involved in drafting the manuscript and revising it critically for important intellectual content. FC: has been involved in drafting the manuscript and revising it critically for important intellectual content. PC: has made substantial contributions to conception and design, or acquisition of data, analysis and interpretation of data and have been involved in drafting the manuscript and revising it critically for important intellectual content. All authors have given final approval of the version to be published.

## Pre-publication history

The pre-publication history for this paper can be accessed here:

http://www.biomedcentral.com/1471-2458/13/486/prepub

## Supplementary Material

Additional file 1 The HFSSM adapted for the SIRS survey (translated back into English). Click here for file

Additional file 2 Food insufficiency questions used in previous French studies. Click here for file
