# Supplementary material for: Prevalence and socioeconomic and geographical inequalities of household food insecurity in the Paris region, France, 2010
Source: BMC Public Health. 2013 May 20;13:486. doi: 10.1186/1471-2458-13-486 (PMC3751527; doi:10.1186/1471-2458-13-486)
Supplement: Additional file 1 — The HFSSM adapted for the SIRS survey (translated back into English). [file 1471-2458-13-486-S1.docx]

**Appendix 1. The HFSSM adapted for the SIRS survey (translated back into English)**

**FS.1a. Can you tell us which of the following statements best describes the situation in your household in the past 12 months, that is, since [current month] of last year?**

*Instruction: Read everything*

**1. Often,** you and the other members of your household didn’t have enough to eat..................................... 🡺FS.1b

**2. Sometimes,** you and the other members of your household didn’t have enough to eat........................... 🡺FS.1b

**3.** In your household, you had enough to eat, but **not always** the kinds of food you wanted........................ 🡺FS.1b

**4.** In your household, you had enough to eat and **always** the kinds of food you wanted............................... 🡺FS.2

**5.** *(Doesn’t know/refuses to answer)*............................................................................................................... 🡺FS.2

**FS.1b. Why was that?**

|  | Yes | No |
| --- | --- | --- |
| Was it because you didn’t have enough money to buy food? | 1 | 2 |
| Was it because you (or someone else in your household) didn’t have enough time to shop or cook? | 1 | 2 |
| Was it because it was too difficult to go to the store? | 1 | 2 |
| Was it because you (or someone else in your household) were (or was) on a special diet? | 1 | 2 |
| Was it because the kind of food you wanted wasn’t available? | 1 | 2 |
| Was it because there weren’t any stores nearby? | 1 | 2 |
| Was it because you (or someone else in your household) were (or was) not able to cook for health reasons? | 1 | 2 |

***TO ALL***

**FS.2. I am now going to read a few statements that may describe a situation that you experienced. Please tell me if these statements were often, sometimes or never true for you and the other members of your household.**

|  | **Often true** | **Sometimes true** | **Never true** | **DK/ RTA** |
| --- | --- | --- | --- | --- |
| In the past 12 months in your household, you were worried that food would run out before you got money to buy more. | **1** | **2** | **3** | **4** |
| In the past 12 months in your household, the food you bought just didn’t last, and there wasn’t any money to buy more. | **1** | **2** | **3** | **4** |
| In the past 12 months in your household, you didn’t have enough money to eat balanced meals. | **1** | **2** | **3** | **4** |

**If there are children under 18 in the household, ask question FS.3. Otherwise, skip to the screen.**

**FS.3. As before, please tell me if the following statements were often, sometimes or never true for you and the other members of your household.**

|  | **Often true** | **Sometimes true** | **Never true** | **DK/ RTA** |
| --- | --- | --- | --- | --- |
| In the past 12 months in your household, you relied on only a few kinds of low-cost food to feed the children because you were running out of money to buy food. | **1** | **2** | **3** | **4** |
| In the past 12 months in your household, you couldn’t serve your children a balanced meal because you couldn’t afford it. | **1** | **2** | **3** | **4** |

| **FILTER**  **If response “3” or “4” to FS.1** |
| --- |
| **or if at least one affirmative response (i.e., “Often true” or “Sometimes true”) to question FS.2**  **or if at least one affirmative response (i.e., “Often true” or “Sometimes true”) to question FS.3, then continue to Stage 2.**  **Otherwise**, **skip to the next stage.** |

**STAGE 2**

**If children under 18 in the household, ask question FS.4.**

**Otherwise**, **skip to question FS.5.**

**FS.4. In the past 12 months in your household, the children weren’t eating enough because you couldn’t afford enough food.**

Often true............. • Sometimes true................. • Never true................. • *(Doesn’t know/Refuses to answer)*

**FS.5a. In the past 12 months in your household, that is, since last [current month], did you ever cut the size of your meals or skip meals because there wasn’t enough money for food?**

• Yes .............................................................. *Go to* **FS.5b**

• No ................................................................ *Go to* **FS.6**

• *(Doesn’t know/refuses to answer)* ............... *Go to* **FS.6**

**FS.5b. How often did this happen?**

• Almost every month .......................................

• Some months, but not every month...............

• Only 1 or 2 months.. ......................................

• *(Doesn’t know/refuses to answer)* .................

**FS.6. In the past 12 months, did you, personally, ever eat less than you felt you should because there wasn’t enough money to buy food?**

• Yes ............................. • No ............................ • *(Doesn’t know/Refuses to answer)*...

**FS.7. In the past 12 months, were you, personally, ever hungry but didn’t eat because you couldn’t afford enough food?**

• Yes ............................. • No ............................ • *(Doesn’t know/Refuses to answer)*...

**FS.8. In the past 12 months, did you, personally, lose weight because you didn’t have enough money for food?**

• Yes ............................. • No ............................ • *(Doesn’t know/Refuses to answer)* ...

| **FILTER** |
| --- |
| **If at least one affirmative response (“YES”, “Sometimes true” or “Often true” ) to any of the questions in Stage 2, then continue to Stage 3.**  **Otherwise**, **skip to the next stage.** |

**STAGE 3**

**FS.9a. In the past 12 months in your household, did you ever not eat for a whole day because there wasn’t enough money for food?**

| Yes 1 🡺 FS.9b |
| --- |
| No 2 🡺 END |
| DK/RTA 3 🡺 END |

**FS.9b. How often did this happen?**

• Almost every month .........................................

• Some months, but not every month..................

• Only 1 or 2 months.... ......................................

• *(Doesn’t know/refuses to answer)*....................
