# Supplementary material for: Prevalence and socioeconomic and geographical inequalities of household food insecurity in the Paris region, France, 2010
Source: BMC Public Health. 2013 May 20;13:486. doi: 10.1186/1471-2458-13-486 (PMC3751527; doi:10.1186/1471-2458-13-486)
Supplement: Additional file 2 — Food insufficiency questions used in previous French studies. [file 1471-2458-13-486-S2.pdf]

Appendix 2 : Food insufficiency questions used in previous French studies

INCA 2 Tool

Which of the following statements best describes the present situation in your household?

1) We always have enough to eat and the kinds of food we want.
2) **We have enough to eat but not always the kinds of food we want.**
3) **Sometimes, we don't have enough to eat.**
4) **Often, we don't have enough to eat.**

Answers 2, 3 and 4 were considered if the answer to the following question was "Yes":

Do you think you don't have the kind of food you want to eat because

[you have an insufficient or irregular inflow of cash and therefore have to cut back on food spending]?

(Answers 2, 3, 4 with "Yes") → Food insufficiency

BSN Tool

Which of the following statements best describes the present situation in your household?

1) We always have enough to eat and the kinds of food we want.
2) **We have enough to eat but not always the kinds of food we want.** → Qualitative food insufficiency
3) **Sometimes, we don't have enough to eat.**
4) **Often, we don't have enough to eat.**

(3 and 4) → Quantitative food insufficiency
